# Interface between women’s health and violence in the training of nurses in Brazil

**DOI:** 10.17533/udea.iee.v39n1e06

**Published:** 2021-03-05

**Authors:** Francisca Tamiris Pereira de Souza, Caik Ferreira Silva, Felice Teles Lira dos Santos Moreira, Regiane Clarice Macedo Callou, Jameson Moreira Belém, Grayce Alencar Albuquerque

**Affiliations:** 1 Nurse, Undergraduate Degree. Regional University of Cariri - URCA, Crato-CE, Brazil. Email: tamirespereira2@hotmail.com Universidade Regional do Cariri Regional University of Cariri CratoCE Brazil tamirespereira2@hotmail.com; 2 Nurse, Master’s Student. Regional University of Cariri - URCA, Crato-CE, Brazil. Email: caik17ferreira@gmail.com Universidade Regional do Cariri Regional University of Cariri CratoCE Brazil caik17ferreira@gmail.com; 3 Nurse, Master. Regional University of Cariri - URCA, Crato-CE, Brazil. Email: felicelira@hotmail.com. Universidade Regional do Cariri Regional University of Cariri CratoCE Brazil felicelira@hotmail.com; 4 Nurse, Master. Regional University of Cariri - URCA, Crato-CE, Brazil. Email: regiane_clarice@hotmail.com Universidade Regional do Cariri Regional University of Cariri CratoCE Brazil regiane_clarice@hotmail.com; 5 Nurse, Master. Regional University of Cariri - URCA, Crato-CE, Brazil. Email: jam.ex@hotmail.com Universidade Regional do Cariri Regional University of Cariri CratoCE Brazil jam.ex@hotmail.com; 6 Nurse, Doctor. Regional University of Cariri - URCA, Crato-CE, Brazil. Professor. Email: geycyenf.ga@gmail.com. Corresponding author Universidade Regional do Cariri Regional University of Cariri CratoCE Brazil geycyenf.ga@gmail.com

**Keywords:** women's health, women, violence, nursing, nursing education, salud de la mujer, mujer, violencia, enfermería, educación en enfermería, saúde da mulher, mulheres, violência, enfermagem, educação em enfermagem

## Abstract

**Objective.:**

To analyze the theoretical interfaces of violence against women in the nursing undergraduate curricula of public Higher Education Institutions in Brazil.

**Methods.:**

Documentary and descriptive study with a qualitative approach. The documentary search happened through the access to the E-mec website for the identification of public Higher Education Institutions for undergraduate nursing degree in Brazil. The menus available online from educational institutions that contained the terms “woman” and “violence” were analyzed. Data processing took place using the IraMuTeQ software, and they were analyzed using the Descending Hierarchical Classification technique.

**Results.:**

The analysis by the software resulted in an important degree of utilization (72.95%), since, of the 244 segments of texts from the menus, 178 were retained. The analysis by the Descending Hierarchical Classification resulted in four thematic categories: Violence against women as a pathological process linked to sexual and reproductive health; Women’s Health: Care, epidemiological, social and cultural aspects; Gender as an analytical category; and Children’s and Adolescents’ Care.

**Conclusion.:**

It was found a connection between the terms “woman” and “violence” to the sexual and reproductive aspects of women (physiological and pathological natures) susceptible to intervention; however, the gender approach is recognized as an analytical category for understanding the vulnerabilities of the female audience to illness and violence.

## Resumen

**Objetivo.:**

Analizar las interfaces teóricas de la violencia contra la mujer en los planes de estudios de pregrado en enfermería de las instituciones públicas de educación superior en Brasil.

**Métodos.:**

Estudio documental descriptivo con abordaje cualitativo. La búsqueda documental se realizó a través del acceso al sitio web E-mec para la identificación de instituciones públicas de educación superior para pregrado en enfermería en Brasil. Se analizaron los menús disponibles en línea de las instituciones educativas que contenían los términos "mujer" y "violencia". El procesamiento de los datos se llevó a cabo mediante el software IraMuTeQ y se analizó mediante la técnica de Clasificación Jerárquica Descendente.

**Resultados.:**

El análisis por el software resultó en un grado importante de utilización (72.95%), ya que, de los 244 segmentos de textos de los menús, se retuvieron 178. El análisis por la Clasificación Jerárquica Descendente mostró cuatro categorías temáticas: violencia contra la mujer como proceso patológico vinculado a la salud sexual y reproductiva; salud de la mujer: aspectos asistenciales, epidemiológicos, sociales y culturales; género como categoría analítica y Atención a la Niñez y Adolescencia.

**Conclusión.:**

Hubo una conexión entre los términos "mujer" y "violencia" con los aspectos sexuales y reproductivos de las mujeres (de naturaleza fisiológica y patológica) sujetas a intervención, sin embargo, el enfoque de género se reconoce como una categoría analítica para comprender las vulnerabilidades de las mujeres a la enfermedad y la violencia.

## Resumo

**Objetivo.:**

Analisar nos currículos de graduação em enfermagem de Instituições de Ensino Superior públicas no Brasil as interfaces teóricas do tema violência contra as mulheres**.**

**Métodos.:**

Estudo de análise documental, descritivo, com abordagem qualitativa. A busca documental ocorreu por meio do acesso ao site E-mec para identificação de Instituições de Ensino Superior públicas de graduação em Enfermagem no Brasil. Foram analisadas as ementas disponíveis *online* das instituições de ensino que continham os termos "mulher" e "violência". O processamento dos dados se deu no *software* IraMuTeQ e foram analisados pela técnica de Classificação Hierárquica Descendente.

**Resultados.:**

A análise pelo *software* resultou em importante grau de aproveitamento (72.95%), visto que, dos 244 segmentos de textos oriundos das ementas, foram retidos 178. A análise pela Classificação Hierárquica Descendente resultou em quatro categorias temáticas: Violência contra as mulheres como processo patológico vinculado à saúde sexual e reprodutiva; Saúde das Mulheres: Aspectos assistenciais, epidemiológicos, sociais e culturais; Gênero como categoria analítica e Atenção à Criança e Adolescentes.

**Conclusão.:**

Constatou-se uma conexão dos termos "mulher" e "violência" aos aspectos sexuais e reprodutivos das mulheres (de cunho fisiológico e patológico) passíveis de intervenção, contudo, a abordagem de gênero é reconhecida como categoria analítica para a compreensão das vulnerabilidades do público feminino ao adoecimento e à violência.

## Introduction

The World Health Organization (WHO) conceptualizes violence as acts sparked off by the intentional use of force or power itself or threatening, against itself, another person or group that entails or results in injury, psychological damage, developmental disability, deprivation and death.(1)

This evil is expressed in different ways, especially in vulnerable individuals, who have their social rights limited or services hindered. In this setting, violence against women is inserted, considered a worrying and complex social and public health problem, taking into account the issue of feminicide, the physical disabilities left in women in situations of violence and the psychological problems with disabling potential. According to Law nº 11.340, known as the Maria da Penha Law, Brazil, domestic and family violence against women is configured as any action or omission based on gender that causes death, injury, physical, sexual or psychological suffering, as well as moral or patrimonial damage, within the scope of the domestic unit, within the family and in any intimate relationship of affection, where the perpetrator lives or has lived with the victim, regardless of cohabitation.(2)

This phenomenon is the result of constructs and gender inequalities established in the several institutions and social relationships between the subjects, who end up being responsible for reproducing patriarchal values, where men take up privileged spaces, thus legitimizing and naturalizing violence against women. Gender conceptions imply the superiority of one sex over the other, especially in private spaces, where the exercise of power by men over women is structured; and, although gender relationships have changed over the years, violence against women still persist in society based on this social dynamic of naturalizing sexual differences to justify the roles assigned to each sex.(3)

In the international setting, the indicators of violence against women are worrying. A study conducted by the WHO on estimates of the prevalence of intimate partner violence against women and sexual violence, with global data from 79 countries and two territories, revealed that almost one third (30%) of all women have suffered physical and/or sexual violence by an intimate partner around the world; and, in some regions, this percentage value reaches 38%; 7% of women have already been sexually assaulted by someone who was not their intimate partner and 38% of all murders of women were perpetrated by intimate partners.(4)

In this setting, it should be emphasized that Brazil occupies a worrying level. According to the Atlas of Violence (2020), in the year 2018, 4519 women were murdered in the country, which represents a rate of 4.3 homicides for every 100 thousand women.(5) The same survey points out that, between 2008 and 2018, there was a 4.2% increase in murders of women; and, in some states, the homicide rate in 2018 more than doubled compared to 2008. A survey held by the DataSenado Research Institute, in partnership with the Observatory of Women against Violence in 2017, with 1116 Brazilian women, showed an increase in the percentage of women who declared that they had been victims of some type of violence perpetrated by a man, and that this percentage went from 18% in 2015 to 29% in 2017, where physical violence was the most perpetrated (67%), followed by psychological (47%), moral (36%) and sexual (15%).(6)

In order to reduce this phenomenon and its impacts, important milestones have been consolidated in Brazil. It should be cited the promulgation of the Maria da Penha Law and the establishment of the National Policy to Combat Violence against Women, which aim to reduce the numbers and sequels of violent acts. The Maria da Penha Law establishes mechanisms to suppress domestic and family violence against women, discussing about its typology, integrated prevention measures, assistance to women in situations of domestic and family violence, assistance by police authorities, urgent protective measures, legal assistance and multidisciplinary team care.(2) The National Policy to Combat Violence against Women aims to establish concepts, principles, guidelines and actions to prevent and confront violence against women, as well as assistance and guarantee of rights, in accordance with international human rights standards and instruments and Brazilian legislation.(7) Nevertheless, in order to consolidate these provisions in the face of violence, intersectoral action is necessary, with an important emphasis on the health sector.(2^,^^7^)

The Brazilian Ministry of Health (MS, as per its Portuguese acronym), in a Technical Cooperation Agreement with the Ministry of Justice, reinforces that health professionals, being in a strategic position to detect and identify risk factors, should be able to diagnose, treat and contribute to the prevention of violence(5); and, in this perspective, it is recommended that health professionals are trained to deal with the different forms of manifestation of this evil and the provision of assistance. Accordingly, the importance of having a training that addresses cross-cutting themes focused on the discussion of violence, health and gender in the undergraduate curricula of health professionals should be emphasized. This condition is necessary, since they feel unable to act in the face of the care towards women in situations of violence, with most of them medicalizing the consequences of this evil, neglect of care and breach of confidentiality.(8) This phenomenon is, in part, the result of the lack of a standardized approach to the theme in the training processes of professionals.

In this context, the nursing category is inserted, composed of nurses, nursing technicians and assistants who, together, constitute a high workforce working in the health field in Brazil and inserted in the Brazilian Unified Health System (SUS, as per its Portuguese acronym) and who are often not prepared to face violence against women. A study conducted with 21 primary health care professionals in Rio Grande do Sul, among them nurses, revealed that there are limitations for the identification of violence against women, among them, silencing, denial and non-recognition of the evil, absence of complaints on the part of women, failures and unpreparedness of the health team to act and fear for the presence of the perpetrator.(8) In another study, conducted with 17 nurses who worked in Primary Health Units in a city in the countryside of Rio Grande do Sul, revealed in a context of violence, the professionals point out limitations such as lack of professional training to face the evil, feeling of unpreparedness, lack of time due to the high workload, difficulties to recognize and deal with violence, low performance of the network care and professional powerlessness.(9)

Such data reveal the difficulty of nurses in assisting women in situations of violence and addressing issues of violence in their daily practices. Thus, the study at stake starts from the following question: does academic training in undergraduate nursing courses in public higher education institutions in Brazil include aspects of the issue of violence against women in their curricular components, in order to recognize and face the problem?

Thus, the aforementioned study aimed to analyze the theoretical interfaces of the issue of violence against women in the nursing undergraduate curricula of public higher education institutions in Brazil. The achievement of this objective will enable the identification of possible gaps and/or strengths of nursing education in this perspective. It should be highlighted that, after identified and corrected possible gaps in this setting, it may contribute to the achievement of the sustainable development goal (SDG) nº 05 - achieving gender equality and empowering all women and girls, listed among the 17 SDGs of the proposed 2030 Agenda by the United Nations.(10) In this sense, actions to reduce/confront violence against women are foreseen, where the systematic integration of the gender perspective in the implementation of the Agenda is crucial. Accordingly, Higher Education Institutions (HEIs) should be committed to responding to the goals of this agenda, through teaching, extension and research actions, since these actions provide the development of qualified human resources for the effective transformation proposed by this agenda.

## Methods

Descriptive study, with documentary design and qualitative approach, conducted in the months of September and October 2019, from the survey of public HEIs in Nursing in Brazil, through access to the E-mec website, an electronic system that makes it possible to follow-up the regulation process of HEIs.(11) The search on the aforementioned website happened through the identification of HEIs (and their amount) that included the nursing course and, later, with an online search on the websites of these educational institutions, seeking to identify the existence of Political-Pedagogical Projects (PPP), curricular matrices and course flowcharts. In the study, it was established as a criterion for the inclusion of HEIs the online availability of said materials on institutional websites, excluding HEIs that did not have them on their platforms. After the identification of these documents, those available online were analyzed for the search for subjects that presented the terms “woman” and “violence”, and those with the presence of both terms were selected for analysis.

In order to extract data from these menus, the authors adopted an instrument that enabled the apprehension of information regarding the location of HEIs, subject, objectives and content covered. After data extraction, the information was organized in the *LibreOffice Writer* program, version 5.3, where the material was prepared through new readings, corrections and decoding of the fixed variable. The exposed encoding concerns the number of universities where the terms searched were found, as can be mentioned *UNE - 01 (...) (University of Nursing or *Universidade de Enfermagem*). The extracted data related to the characterization of HEIs were tabulated in *Microsoft Office Excel* 2010 spreadsheets and presented under simple descriptive analysis, in absolute and relative numerical values. The processing of the menus was performed using the *Interface de R pour les Analyses Multidimensionnelles de Textes et de Questionnaires* (IRaMuTeQ) program, version 0.7, alpha 2. This free software allows different forms of statistical analysis of texts, produced from documents, among others.(12)

For this study, the Descending Hierarchical Classification (DHC) was used, which divides the corpus into classes, grouping the words according to the greatest association with the class and presenting the percentage of representation in the studied corpus. To that end, the programmatic content of the menus was processed in IRaMuTeQ. The processing in that program made it possible from the DHC dendrogram to design four thematic categories. With regard to research involving documents/files of institutions and/or secondary databases containing only available and public domain data, that do not identify research participants and the non-involvement of human beings, there is no need for approval from the Research Ethics Committee (CEP, as per its Portuguese acronym) and the National Research Ethics Commission (CONEP, as per its Portuguese acronym).

## Results

In order to enable a better understanding, the data analyzed were organized in a table; and from this, the quantitative profile of public HEIs in nursing undergraduate courses was outlined. In this search, the nursing course was active in 87 public HEIs, distributed over 148 campuses; and, of these, 10 contained licentiate courses and 138 bachelor courses, with one course with both modalities, as displayed in Table 1.

**Table 1:** HEIs with Nursing courses in available on the E-mec portal. Brazil, 2019

Region	nº HEIs	%	nº of campus	%
Northeast	27	31.0	60	40.5
North	11	12.6	19	12.8
Midwest	8	09.1	18	12.1
South	19	21.8	22	14.8
Southeast	22	25.2	29	19.5
Total	87	100	148	100

The Northeast region of the country has the largest number of public universities with a nursing course, which is also equivalent to the highest number of campuses. PPPs were available online in 73 courses, curriculum matrices in 57 and flowcharts in five. Of this total, 73 subjects were identified with the terms “woman” or “violence”, with 63 available online. Of this amount, only 35 together contained the aforementioned terms in their content. At the end of the searches, no material available online was found in 18 institutions. Accordingly, the description of the results presented by IRaMuTeQ was only possible from the processing of the 35 menus of the nursing courses that contained the terms “woman” and “violence”. The analysis through the software resulted in a considerable degree of utilization (72.95%); since, of the 244 text segments from the menus, 178 were retained.

**Figure 1:**
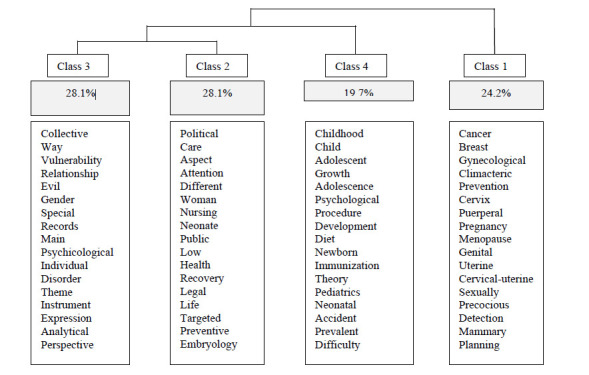
Descending Hierarchical Classification with partitions and corpus content of the research for the Nursing course in Brazil. E-mec, 2019

The Descending Hierarchical Classification (DHC) for the nursing course divided the textual corpus into four classes, with the words from classes *p*<0.0001. Firstly, the corpus was divided into two subcorpora. In a second moment, there was a new subdivision, and class four was subdivided into classes two and three. The most expressive classes were classes two and three, with 28.2% representation, followed by class one (24.2%) and class four (19.7%), as displayed in Figure 1.

From the identification of the classes and their prominent words, the qualitative data processing enabled us to extract the way in which the teaching of the themes “women’s health” and “violence” happens in the training processes of professional nurses in public HEIs in Brazil, which supported the development of four thematic categories.

### Class 1 - Violence against women as a pathological process linked to sexual and reproductive health

In the menus analyzed that make up class 1, it is identified that the presence of the terms “violence” and “women’s health” was associated with teaching about nursing care to pathological disorders linked to sexual and reproductive health, with emphasis on the gynecological problems, such as sexually transmitted infections: (...) **prevention** and **treatment** of STIs and **AIDS**, nursing in gynecology, **cervical and breast cancer**, **gynecological**, endocrine and genetic alterations, care towards **women** victims of violence, climacteric **care** (. ..) (UNE_03, Score: 331.46); (...) coping with violence against **women**, implications for **reproductive** health, **gynecological problems**, abdominal and **pelvic** pain, vulvar itching, **genital** discharge, **genital** bleeding, **breast** pain **(*mastalgia*)**, **breast** lump, nipple discharge, dysmenorrhea, **pre-menstrual** and inter-menstrual syndrome, **pelvic** and abdominal tumors (...) (UNE_36, Score 182.02); (...) health-disease process, **gynecological** and **reproductive** health care, sexuality and gender, **climacteric** and **menopause**, **reproductive planning**, violence, nursing **care** systematization, nursing process, nursing classification systems, **gynecological** consultation, diagnostic support, applied pharmacology (...) (UNE_16, Score: 153.40).

### Class 2 - Women’s Health: Care, epidemiological, social and cultural aspects

With greater representativeness in this study, class 2 reveals that the teaching of the themes “violence” and “women’s health” was associated with the recognition of health determinants and epidemiological aspects related to assistance (care), care protocols and ethical and legal bases of nursing care in the health-disease process, especially sexual and reproductive ones: (...) sexual and **reproductive** rights, violence against **women**, epidemiology, legal **bases** and **assistance (care)**, **health care protocols for women**, **nursing care process** in prevention, promotion and **recovery of women’s health** at **different stages of life** (...) **(UNE_09, Score 128: 32);** (...) **legal bases** of **nursing** practice in family planning for **women** and the collective space, emphasis on the social **role** of gender and work, sexual and **reproductive** rights, intra-family violence, **cultural** and **ethical** aspects of care for **women in Brazilian society** (...) **( UNE_31, Score 110.94)**; (...) study of the **epidemiological** and **determining** aspects of the **health-disease** process of women, violence against **women** and maternal mortality, **nursing** actions in the care of **women** in the **reproductive** process and **pre-conceptional** consultations and in gynecological disorders (...) (UNE_07 , Score 106.66); (...) **women’s roles in society** and their repercussions on **life** and the **reproductive** process, **determinants** of maternal and perinatal morbidity and mortality, violence against **women**, **female reproductive cycle**, development and hormonal action, pathologies related to the female reproductive system (...) **(UNE_29, Score: 46.17)**.

### Class 3 - Gender as an analytical category

Class 3 highlights that the approach between the themes “violence” and “women’s health” happened from the recognition of the category related to gender as necessary for the analysis of health determinants. The historical and cultural perceptions of gender issues are outlined in nursing education, being assumed as an analytical category for understanding the vulnerabilities of the female population, with emphasis on violence: (...) **gender and collective health**, **critical** analysis of the **socio-historical construction of gender relationships** in society in **health** and nursing, **gender as an analytical category** for **understanding** power **relationships**, **violence, vulnerability, health needs** and the **health-disease** process (...) **(UNE_25, Score: 178.49);** (...) **vulnerability, gender and violence**, **themes** to talk about in **health**, aiming to **discuss** the **theoretical constructions** of **violence, vulnerability and gender**, thus seeking to dialogue with the **health field**, **especially** with the assumptions of family **health** (...) ( **UNE_26, Score: 84.23);** (...) society, **health and violence**, human rights and **health**, **expressions** of **violence** in society, culture, gender, **race** and ethnicity, **repercussions** of **violence** on health in daily life, **needs** and possibilities for **professional intervention** in cases of **violence** (...) **(UNE_28, Score: 66.43).**

### Class 4 - Children’s and Adolescents’ Care

In the last category, referring to class 4, with less representation in this study, it is clear that the teaching of the themes “violence” and “women’s health” happened through discussions regarding vulnerabilities to evils, focusing on children and adolescents: (...) **growth** and **development**, **physical, biological** and **psychosocial characteristics**, sexual development, **psychological** approach of **children** and **adolescents**, nursing consultation for **children** and **adolescents**, **pregnancy during adolescence**, **accidents** and violence during **childhood** and **adolescence (...)** (UNE_05, Score: 331: 46); (...) nursing consultation for **children** and **adolescents**, **pregnancy during adolescence**, **accidents** and violence during **childhood** and **adolescence**, **illegal drugs**, **hospitalized children and adolescents**, nursing **procedures**, **high-risk newborns** (... ) (UNE_12, Score: 303.06); (...) process of caring for **newborns**, breastfeeding and **child nutrition**, domestic violence during **childhood** and **adolescence**, **child growth**, **child development**, aspects of vaccination in the health of **children** and **adolescents** (...) (UNE_34, Score: 284.67).

## Discussion

The acquisition of skills/abilities by students in the health area during academic training is reflected in qualified professional profiles and sensitive to the population’s health-disease process. Thus, students in this area need to be trained to deal with the specificities and demands of different social segments, among which the female audience stands out. National Health Survey held by the Brazilian Institute of Geography and Statistics in 2013, in Brazil, revealed that, in view of the demand for health services, women are the majority,(13) in part, as a result of the historical and cultural socialization of care for the other and yourself due to gender issues.

Given this reality, which demonstrates that women constitute the largest number of users of health services, as well as being more susceptible to certain evils, such as violence, due to gender issues, it should be emphasized the university’s commitment and responsibility to provide an academic education contemplating the specificities of this audience and the approach of transversal themes, among them, violence, gender and sexuality in health courses, such as nursing. Thus, in view of the analysis of the menus available online that contained the terms together “woman” and “violence”, the low amount of available materials stands out, although regulatory guidelines reinforce just the contrary. In Brazil, Decree nº 9.235, dated December 25^th^, 2017, provides for the exercise of the functions of regulation, supervision and evaluation of higher education institutions;(14) and, in its sole paragraph, establishes that it will be up to HEIs to disseminate their institutional acts, courses and pedagogical documents to students, under the terms of article 47 of Law nº 9.394, dated 1996.

Although in view of the difficulty of providing educational materials by HEIs, compared to the material analyzed, there is an approach to violence against women in association with the pathological conditions of the sexual and reproductive system and its implications on this, signalizing a type of nursing care directed to the resolution of problems susceptible to a specific and biologicist intervention, as shown in class 1 of this study. As for class 2, the approach of aspects related to the determinants of the health-disease process is highlighted, which increase vulnerabilities to illness in women, especially those associated with sexual and reproductive health, with the inclusion of the term “violence against women” presented as a topic resulting from cultural aspects (gender) and responsible for illness, mainly in the sexual and reproductive dimension. In this sense, nursing care is directed in order to avoid further complications; and, for this purpose, in addition to teaching care protocols, cultural, ethical and legal aspects should be addressed.

With regard to class 3, the menus discuss gender from a historical and social perspective, associating the concept as a factor responsible for female vulnerability to illness and forms of discrimination, oppression and violence, both symbolic and material, thus providing a new reading scheme of social phenomena, which implies greater possibilities of professional intervention in the health field, with greater emphasis on the fields of collective health/family health. Finally, in class 4, it is noted that aspects referring to nursing care for children and adolescents stand out, especially focused on the milestones of biopsychosocial growth and development and its most frequent evils, considering the adolescent’s health, the approach, in most cases, aimed at the female audience, which is more susceptible to violations of sexual and reproductive rights and, therefore, more susceptible to complications, such as violence and pregnancies.

Accordingly, in general, nursing approaches stand out in the issues related to women’s sexual/reproductive health, followed by the performance of nursing professionals in the face of physiological and pathological aspects in life cycles. It is noteworthy that this evidence is also found in other pertinent literature. A review study, complemented by interviews with health professionals, revealed that the care for women is still reduced to narratives guided by biological-clinical and technical-scientific aspects, thus legitimating the health professional as the authority in guiding and directing care over the female body and establishing its conception as true by disregarding women’s autonomy and knowledge.(15)

Despite the approach directed to physiological and pathological aspects, the teaching of ethical and legal aspects of nursing professionals and the duties of nurses in the care of women also stand out with a focus on social determinants for understanding the health-disease process. In this sense, in order to enable future professionals to provide nursing care including the guidelines of the National Policy for Comprehensive Care for Women’s Health(16) (PNAISM, as per its Portuguese acronym), it appears that the nursing courses that have been analyzed seek to guarantee qualification of future nurses through the approach/teaching of themes that corroborate with the policy guidelines, with the epidemiological profile of the evils, as well as with the responses to the 2030 Agenda - of the Sustainable Development Goals.

PNAISM consolidates the fields of women’s sexual and reproductive rights, with emphasis on improving obstetric care, family planning, care for unsafe abortion and fight against domestic and sexual violence. This policy was formulated based on the evaluation and gaps of the predecessors as assistance (care) to women in climacteric/menopause; gynecological complaints; infertility and assisted reproduction; women’s health during adolescence; chronic-degenerative diseases; occupational health; mental health; infectious-contagious diseases and care towards rural, disabled, black, indigenous, prisoners and lesbian women.(16) Taking into consideration PNAISM care objectives, it can be noticed that, although teaching and assistance to women’s health in higher nursing courses are mostly focused on sexual and reproductive physiological and pathological processes, when the approach to the “violence against women” is analyzed, the discussion on gender issues in the sexual and reproductive care of this audience is inferred. Addressing gender issues in PNAISM implies taking as a reference the patterns of life through which men and women relate in their daily lives as a result of a social/cultural determination, which affects the health-disease-care process of women.(16)

This approach stands out in class 3 of this study, by understanding gender as an analytical category that implies the determination of worsening and vulnerabilities of women to illness. Recognizing gender, class and race/ethnicity inequalities as social determinants of feminine illness and vulnerability to violence, allows us to think about public policies in the dimension of collective health, as well as promotion and quality of life. In the face of nursing education, the recognition of gender issues implies the training of professionals capable of understanding that power relationships and their social manifestations interfere with the health-disease process of the female audience, thus exacerbating social inequities and increasing the susceptibility of this population to physical and mental illness and other evils, such as violence.

Gender-based violence is present in the culture of all countries, regardless of their degree of development, and is expressed to a greater or lesser extent. Culturally, it is reproduced through ill-considered, historically and socially learned behaviors in social institutions such as the Church, school, family and the State, which directly contribute to male sovereignty and female oppression.(17) Historically, it can be argued that women have been socially oppressed according to the impositions of gender(3) that are maintained by social institutions contributing to disseminate the idea that the female gender is inferior and endowed only with protective instincts. Addressing gender issues in curricula still implies a potential reduction in the number of cases of institutional violence in health services targeted at women, often perpetrated by health professionals and materialized in the face of disregarded and medicalized complaints from women who suffer domestic/inter-personal violence and obstetric violence.

It is known that cases of institutional violence can drive women away from the health services in violent situations, which makes it difficult to intervene early in the face of the phenomenon and reduce its sequels. In most cases, when seeking assistance in health services, women in violent situations go to hospital institutions, due to their physical impairments; however, this service is often considered a gateway to the health system for meeting cases resulting from the evil in question.

In a study(18) that evaluated the low number of reported cases of violence by the Family Health Strategy (ESF, as per its Portuguese acronym), it was noted that these rates are justified by the unpreparedness of professionals in the recognition of the evil. In Greater São Paulo, a study conducted in 19 health services revealed that only 3.8% showed records of the situation of violence suffered by women in a total of 3,193 medical records analyzed. This absence of records demonstrates the invisibility and non-recognition of violence as an object of intervention in the health field.(19) Corroborating this reality, another study(20) with 42 professionals (nursing technicians, nurses and physicians) conducted in a municipality of the state of Santa Catarina, Brazil, revealed that the deficit of professional preparation, either during undergraduate courses or in health services, leads, many times, to a superficial reception with unqualified assistance (care) to women in situations of violence. Moreover, the absence of holistic attention in the care of women in situations of violence implies the non-recognition of factors associated with the occurrence of violence and its consequences, with capillarization to the family, following the example of the offspring. Nonetheless, despite its weaknesses, the health sector has great potential for addressing violence, since, in these services, the performance of professionals is decisive and can contribute to the identification of this evil, as well as offering prevention, attention and development of studies in face of the problem.

In fact, the sequels of violence against women are not restricted only to women, but they gain capillarity within the family scope, and perhaps this condition can justify the identification of the approach of the term “violence” associated with other phases of the life cycle, with emphasis on children’s and adolescent’s health. A study(21) conducted with 239 adolescents from a public school in a peripheral neighborhood of the city of Salvador, Bahia, when analyzing the prevalence and factors associated with violence in this audience, pointed out that the prevalence of intra-family violence among students was 60.67%.

In this age group, another factor that justifies addressing the term “violence” is the greater susceptibility that children and adolescents have in relation to victimization. Given its peculiar stage of development, this audience is pointed out as more vulnerable to the evil, especially in the face of lower socioeconomic levels and when individuals are female.(22) In face of the latter, about 40 million children and adolescents, mainly girls, suffer sexual abuse annually.(23)

In Brazil, sexual violence occupies the second highest type of violence among individuals in the 10-14 age group, with physical violence in first place.(24) This is a form of violence that is not fully recognized as a public health problem and requires strategies by governments for identification and reporting, since most abused adolescents within families are at high risk of developing a series of disorders(24) Thus, in face of the complexity of violence and its implications, a discussion and reflection on this issue is required in the training of future health professionals; and, in face of nursing, the need to sensitize this group to an ethical, moral and legal performance. Especially in the face of adolescence, the recognition of the approach of this phenomenon in the menus directs the future nursing professionals for early interventions, with high potential for reduction of sequels and consequences of violence both now and in the future. It is reinforced that the precocious contact with professionals and health services is indispensable for an agile and efficient approach in the reception and management of this demand and technical standards of MS and of the board councils that orient professionals and regulate ethical, legal and moral standards for nursing performance in the face of this evil.

In addition to the guaranteed care, the legal conducts indicate that it is the duty of the health professional to notify confirmed or suspected cases of violence. The pertinent literature has attributed the underreporting of violence to the lack of knowledge on the part of health professionals about their legal responsibility. Although it is a compulsory notification event, a study(25) points out that, even in face of the obligation to register the phenomenon, such procedure is still invisible in the routine of health professionals, where under-notification is correlated to the absence of technical and scientific information on the subject, as well as factors such as lack of knowledge about the obligation of notification, fear of reprisals from the perpetrator, embarrassment to question details of the violence or trivialization of facts.

Facing this scenario and the obligation of all health services to provide qualified care to women in situations of violence, the context imposes broad debates on the proposal to institute the nurse’s role in the curricula, in order to cope with the phenomenon. Therefore, it is crucial for HEIs to insert aspects aimed at teaching violence against women in their training processes, for its recognition and confrontation with the purpose of its prevention, fostered by the collaboration among academic communities and practices among university students, teachers and professionals.

As final considerations, violence against women is a public health issue and, therefore, it is necessary to work on this issue during professional training in health, with emphasis on nursing staff, the largest health professional body in Brazil, so that the confrontation of this evil can imply the prevention and reduction of its indicators. Thus, during the analysis of the presence of the terms “woman” and “violence” on the menus obtained from nursing undergraduate courses, it was found, in most cases, a connection between these terms and the sexual and reproductive aspects of women (physiological and pathological) that can be addressed, although the gender approach is recognized as an analytical category for understanding the vulnerabilities of the female audience to illness and violence.

Furthermore, the study allowed the identification of gaps in nursing education in the face of violence perpetrated against the feminine audience, since it was not identified the approach of themes related to the network of confrontation of the violence against women, itineraries in search of assistance and critical routes of women in situations of violence, for example, in the material analyzed. These are important contributions of the research, which may be discussed and analyzed by the academic community with the purpose of changing this scenario.

Although important, this study has shown limitations, where the lack of online material from HEIs is the main one. It is clear that the limitation of full access to documents that support teaching in undergraduate nursing courses make it difficult to conduct research that seeks, in documents of these institutions, responses to social questions. Although we are aware of this limitation, this work was intended to raise awareness for the promotion of discussions that may corroborate with a humanized practice in health services and promote qualified assistance (care) to women in situations of violence. Moreover, it is suggested the importance of going ahead in this research *in locus* in HEIs, as well as deepening the study and the information provided by students and teachers.
